# FOXL2 Is an Essential Activator of SF-1-Induced Transcriptional Regulation of Anti-Müllerian Hormone in Human Granulosa Cells

**DOI:** 10.1371/journal.pone.0159112

**Published:** 2016-07-14

**Authors:** Hanyong Jin, Miae Won, Si Eun Park, Seunghwa Lee, Mira Park, Jeehyeon Bae

**Affiliations:** 1 School of Pharmacy, Chung-Ang University, Seoul, Korea; 2 Department of Pharmacy, CHA University, Seongnam, Korea; 3 Department of Life Science, Chung-Ang University, Seoul, Korea; Baylor College of Medicine, UNITED STATES

## Abstract

Anti-Müllerian hormone (AMH) is required for proper sexual differentiation by regulating the regression of the Müllerian ducts in males. Recent studies indicate that AMH could be an important factor for maintaining the ovarian reserve. However, the mechanisms of AMH regulation in the ovary are largely unknown. Here, we provide evidence that *AMH* is an ovarian target gene of steroidogenic factor-1 (SF-1), an orphan nuclear receptor required for proper follicle development. FOXL2 is an evolutionally conserved transcription factor, and its mutations cause blepharophimosis, ptosis, and epicanthus inversus syndrome (BPES), wherein affected females display eyelid defects and premature ovarian failure (POF). Notably, we found that functional FOXL2 is essential for SF-1-induced *AMH* regulation, via protein–protein interactions between FOXL2 and SF-1. A BPES-inducing mutant of FOXL2 (290–291delCA) was unable to interact with SF-1 and failed to mediate the association between SF-1 and the *AMH* promoter. Therefore, this study identified a novel regulatory circuit for ovarian AMH production; specifically, through the coordinated interplay between FOXL2 and SF-1 that could control ovarian follicle development.

## Introduction

Anti-Müllerian hormone (AMH), also known as Müllerian inhibiting substance, is a member of the transforming growth factor β (TGF-β) family. This is known to be an essential hormone for proper sexual differentiation as it regulates the regression of the Müllerian ducts in males [1]. In recent years, a new functional role for AMH in ovarian follicle development has been reported based on studies using *Amh* knockout mice; these animals display infertility associated with the early depletion of the follicle pool [2]. AMH is highly expressed in the granulosa cells of small, growing follicles of the ovary [3]. In women, serum AMH levels decrease with age, leading to undetectable levels during menopause [4]. In addition, serum AMH levels in patients with primary ovarian insufficiency (POI) or premature ovarian failure (POF) are extremely low or undetectable [5, 6]. Together, these findings imply that AMH might be an important hormone for maintaining the ovarian reserve. However, regulatory mechanisms of ovarian AMH production remain largely unknown.

Steroidogenic factor-1 (SF-1), also known as NR5A1, is an orphan nuclear receptor that has multiple functions in reproduction, steroidogenesis, and sexual differentiation [7]. SF-1 consists of two zinc finger DNA-binding domains, a ligand-binding domain, and a hinge region in between [8]. *Sf-1*-deficient mice exhibit adrenal and gonadal development abnormalities [9, 10]. In addition, female mice with a conditional *Sf-1* knockout in granulosa cells display infertility associated with decreased follicle numbers [11]. SF-1 regulates the transcription of genes crucial for reproduction, including *AMH*, *DAX-1*, steroidogenic acute regulatory protein (*StAR*), cholesterol side chain cleavage enzyme (*SCC*), *CYP17*, and *CYP19* (*aromatase*) [12–16]. The effect of interplay between SF-1 and SOX8, SOX9, Wilms’ tumor 1 (WT1), DAX-1, GATA4, and NF-κB on the transcription of *AMH* in Sertoli cells during testis development is relatively well defined [17–22]. However, to the best of our knowledge, the role of SF-1 in AMH regulation in the ovary remains unknown.

FOXL2 is an evolutionally conserved transcription factor that belongs to the forkhead family [23]. This family of genes encodes key molecules that regulate fundamental functions, including apoptosis, differentiation, proliferation, metabolism, and developmental processes [24]. In humans, *FOXL2* mutations cause blepharophimosis-ptosis-epicanthus inversus syndrome (BPES; OMIM #110100), which is an autosomal dominant disease, and affected individuals display eyelid defects with or without POF [25]. Female, *FoxL2*-deleted, homozygous mice are infertile due to the premature exhaustion of ovarian follicles [26, 27]. FOXL2 ablation in the ovary of adult mice induces somatic sex reprogramming of ovaries to testes [28]. These findings underscore the crucial role of FOXL2 in the ovary. Recently, we identified that *AMH* is a target gene of FOXL2, and that depletion of AMH accelerates follicle growth, which could be effectively overcome by FOXL2 overexpression in mouse ovaries [29]. We also reported that FOXL2 interacts with SF-1 in granulosa cells [30].

In the present study, we investigated the inter-regulatory network of three key molecules (AMH, FOXL2, and SF-1) crucial for controlling the ovarian reserve. Here, we present, for the first time, direct experimental evidence indicating that *AMH* is a target gene of SF-1 in human granulosa cells. Furthermore, we identified a critical regulatory role for FOXL2, which acts as an essential factor for the transcriptional regulation of *AMH* by SF-1.

## Materials and Methods

### Cell culture and transfection

Human, adult-type granulosa cell tumor (GCT)-derived KGN cells (Drs. Yoshihiro Nishi and Toshihiko Yanase, Kyushu University, Fukuoka, Japan) and juvenile-type GCT-derived COV434 cells (Sigma-Aldrich, St. Louis, MO, USA), and human kidney-derived 293T (American Type Culture Collection, Rockville, MD, USA) cells were cultured in Dulbecco’s modified Eagle’s medium (DMEM) or DMEM-F12 containing 10% fetal bovine serum (FBS) and 1% penicillin–streptomycin. All cells were transfected with Lipofectamine® 2000 (Invitrogen, Carlsbad, CA, USA) according to the manufacturer’s instructions.

### Plasmid construction

Generation of constructs, Myc-tagged FOXL2, FLAG-tagged SF-1, human *AMH* promoter, and its FOXL2-binding elements (FBE) mutants was described previously [29, 30]. Constructs driving the expression of Myc-tagged mutated FOXL2 (290–291delCA) were generated by recombinant PCR using the following primers: FOXL2 wild type (WT)-F (5′-CTAGAATTCAAATGATGGCCAGCTACCCC-3′) with 290–291delCA-R (5′-CCGGTCTCGGGCCAAGCAG-3′); 290–291delCA-F (5′-GAGACCGGTCGCACA-3′) with FOXL2 WT-R (5′-CATTCGCGCCTCGATCTCTGACTCGAGTAG-3′). The PCR products were digested with *Eco*RI and *Xho*I (Takara Bio, Otsu, Shiga, Japan) and ligated into the pCMV-Myc vector (Clontech, Mountain View, CA, USA).

### Luciferase reporter assay

KGN and COV434 cells (2 × 10^5^) were transfected with 600 ng of *AMH*-luciferase reporter, 100 ng of pCMV β-galactosidase (Clontech), in addition to the indicated plasmids (300 ng) as well as small interfering nucleotides (200 nM) using Lipofectamine 2000 (Invitrogen) according to the manufacturer’s instructions. Cells were then incubated in 12-well plates containing medium for 24 h. Luciferase activity was assessed as previously described [30]. The absorbance was measured with the FlexStation3 Microplate Reader (Molecular Devices, Sunnyvale, CA, USA).

### Small interfering RNAs

Small interfering RNA (siRNA) target sequences specific to FOXL2 and SF-1 were 5′-GCUCCUGUCGCUCCUCUUU-3′ and 5′-GUCUGCCUCAAGUUCAUCA-3′, respectively. The control siRNA sequence used was 5′-CCUACGCCACCAAUUUCGU-3′. The sense and antisense oligonucleotides were annealed in the presence of annealing buffer (Bioneer, Daejeon, South Korea).

### Immunoblotting and immunoprecipitation

KGN cells were transfected with the indicated plasmids and specific siRNAs. Twenty-four hours after transfection, cell lysates were prepared for immunoprecipitation with antibody-linked protein G Dynabeads® (Invitrogen) according to the manufacturer's instructions. After incubation, the samples were boiled and subjected to SDS-PAGE and immunoblotting with appropriate antibodies. The membranes were imaged using a ChemiDoc™ XRS+ System Imager (Bio-Rad Laboratories Inc., Hercules, CA, USA). The following antibodies were purchased and used in this study: anti-AMH (AP9940c; Abgent, San Diego, CA, USA), anti-SF-1 (sc-28740; Santa Cruz Biotechnology, Santa Cruz, CA, USA), anti-GAPDH (sc-25778; Santa Cruz Biotechnology), anti-PARP (sc-74469; Santa Cruz Biotechnology), and control rabbit IgG (sc-2027; Santa Cruz Biotechnology), anti-α-tubulin (LF-PA0146; AbFrontier Seoul, Korea), anti-c-Myc (631206; Clontech), anti-FLAG (2368) (Cell Signaling, Danvers, MA, USA), and anti-FLAG (F1804; Sigma-Aldrich). The polyclonal rabbit FOXL2 antibody was described previously [30].

### Subcellular fractionation

KGN cells were transfected with the indicated plasmids as well as siRNAs for 24 h and fractionation of nuclear and cytosolic compartments was performed as previously described [30].

### Electrophoretic mobility shift assay (EMSA)

EMSA was performed as previously reported [30]. The probe was designed based on known SF-1 binding sequences (SBEs) at -92 to -84 and -218 to -210 [21]. Double-stranded oligonucleotides were generated based on the following human *AMH* sequences: 5′-TGTCCCCCAAGGTCGCGGCAG-3′ or 5′-CTGCCGCGACCTTGGGGGACA-3′T oligonucleotides were annealed before use.

### Chromatin immunoprecipitation–quantitative polymerase chain reaction (ChIP–qPCR) analysis

ChIP assays were performed as previously described [30]. DNA was amplified using primer sets flanking the putative SF-1 binding element 1 (SBE1; -134/-6) and SF-1 binding element 2 (SBE2; -293/-133) of the *AMH* promoter as follows: forward (5′-GAAGGCCACTCTGCCTGGAGT-3′) with reverse (5′-GGGCTGGGCTGCCTGCCTTAA-3′); forward (5′-CAGCGCTGTCTAGTTTGGTT-3′) with reverse (5′-TCTCAAAGAGCCCTTTCTGT-3′). The primers of the FOXL2-binding motifs (FBE1 and FBE2) in the *AMH* promoter were described in our previously study [29]. Products were analyzed by quantitative real-time PCR.

### Recombinant protein purification

SF-1 as well as WT and mutated (MT; 290–291delCA) FOXL2 were purified from mammalian cells after transfection of 293T cells with pcDNA3-FLAG-NR5A1 (FLAG-tagged SF-1) as well as Myc-tagged WT and MT FOXL2 plasmids. Twenty four hours after transfection, the cells were lysed in RIPA buffer containing 1 mM Na_3_VO_4_, 10 mM NaF, and a protease inhibitor cocktail, and were frozen at −80°C for 2 h. Following incubation, the supernatant was separated from 293T cell lysates and incubated with BS3-crosslinked anti-FLAG or anti-Myc-Dynabeads protein G (Invitrogen) overnight, at 4°C. The antibody–protein G crosslinked Dynabeads were washed with precooled PBS-T three times and protein G Dynabead-linked proteins were eluted with 0.1 M glycine buffer (pH 2.0) for 1–2 min, and immediately neutralized with 1 M Tris buffer (pH 10.0).

### Immunofluorescence analysis

KGN cells (2 × 10^4^) were seeded onto round coverslips in 24-well plates. At 24 h post-transfection, cells were fixed with 4% paraformaldehyde for 15 min at room temperature. The fixed cells were permeabilized with 0.2% Triton-X 100 in TBS for 15 min and then incubated with 2% BSA in TBS containing 0.1% Tween 20 (TBS-T) for 2 h. Fixed cells were incubated for 12 h with primary antibodies in TBS-T. Anti-Myc (1:100) and anti-SF-1 (1:100) antibodies were used to detect the localization of FOXL2 and SF-1, respectively. After washing three times with TBS-T, the cells were stained with Alexa Fluor® 488 goat anti-mouse IgG (1:1000) (Invitrogen) and Alexa Fluor® 546 goat anti-rabbit IgG (1:1000) (Invitrogen) for 1 h. After additional washing with TBS-T, the coverslips were mounted on slides using mounting solution with DAPI. Fluorescence was detected using a Zeiss LSM 510 META confocal microscope (Carl Zeiss, Göttingen, Deutschland).

### Statistical analysis

Data analysis was performed using SAS version 9.2 (SAS Institute, Cary, NC, USA). Statistically significant differences were identified using either the Student–Newman–Keuls multiple range test or the Fisher’s least significant difference test, at the 5% level of significance.

## Results

### SF-1 transactivates ovarian *AMH* in a FOXL2-dependent manner

**Fig 1:**
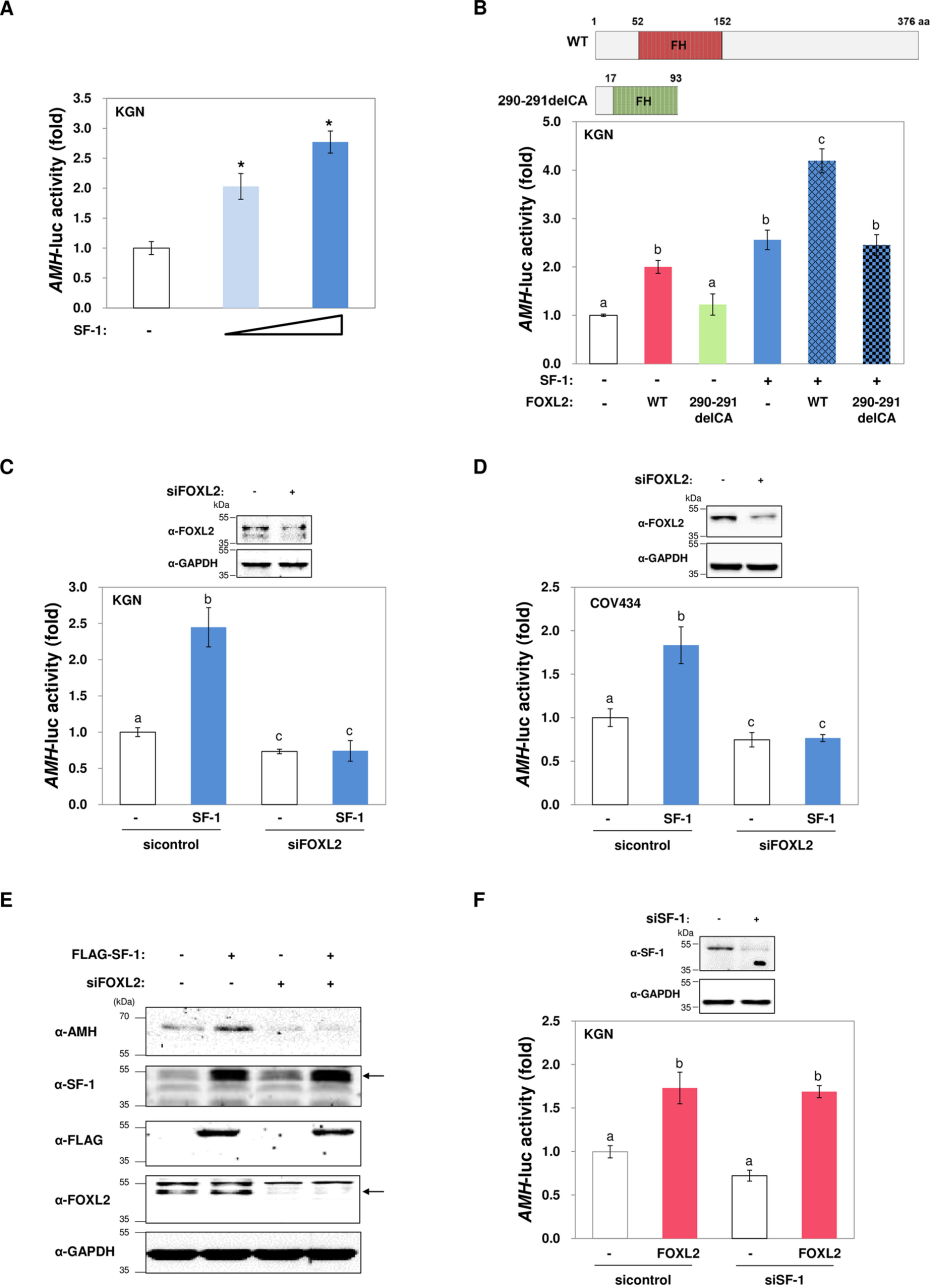
Transcriptional activation of *AMH* by SF-1 requires functional FOXL2 in human granulosa cells. (A) *AMH* promoter activation by SF-1 was determined by luciferase assays after transfection of KGN cells with increasing amounts of plasmids (150 and 300 ng) encoding FLAG-tagged SF-1. The luciferase activity was analyzed 24 h after transfection. (B) Secondary structures of wild type (WT) and truncated mutants of FOXL2 (290–291delCA) are illustrated. The *AMH* promoter constructs were co-transfected with 300 ng of FLAG-SF-1 and Myc-tagged WT FOXL2 or mutant FOXL2 (290–291delCA) in KGN cells. Cells were harvested for luciferase assays 24 h after transfection. (C–D) KGN cells (C) or COV434 cells (D) were transfected with control or SF-1 plasmids together with control siRNA or specific FOXL2 siRNA. *AMH* luciferase activity was analyzed 24 h after transfection. Efficient silencing of FOXL2 using a specific siRNA (200 nM) was confirmed by western blot analysis. (E) Expression of AMH, after overexpression of FLAG-tagged SF-1 in either FOXL2-knockdown or control siRNA-transfected KGN cells, was determined by immunoblot analysis using the indicated antibodies. Arrows indicate the expected position of the proteins. (F) KGN cells were transfected with control or FOXL2 plasmids together with control siRNA or a specific SF-1 siRNA. *AMH* luciferase activity was analyzed 24 h after transfection. Efficient silencing of SF-1, using specific siRNA (200 nM), was confirmed by western blot analysis. GAPDH was used as a loading control. Data (means ± SEM) from all promoter assays were obtained from at least three independent experiments, each conducted in triplicate, and are presented as a fold increase of relative luciferase units compared to controls. Asterisks or different letters indicate statistically significant values compared to the control (*p* <0.05).

To understand the regulatory function of SF-1 on AMH in the ovary, the effect of SF-1 on transcriptional regulation of the *AMH* promoter was determined after ectopic expression of SF-1 in the human, GCT-derived KGN cell line. As shown in Fig 1A, SF-1 clearly activated the *AMH* promoter in granulosa cells. Overexpression of WT FOXL2 also transactivated *AMH* (Fig 1B), as we previously reported [29]. In contrast, a FOXL2 mutant harboring a dinucleotide deletion (290–291delCA), detected in a patient with BPES type I who exhibited POF [31], failed to elicit transactivation of *AMH* (Fig 1B). Co-expression of WT FOXL2 further potentiated SF-1-induced *AMH* activation, but the FOXL2 mutant did not (Fig 1B). Furthermore, SF-1-induced *AMH* transcriptional activation was completely abolished in FOXL2-silenced KGN cells (Fig 1C). A similar effect was observed in COV434 cells, another type of human granulosa cell line (Fig 1D). Regulation of AMH by SF-1 and FOXL2 was further confirmed by immunoblot analysis; herein, the level of AMH protein increased after SF-1 overexpression, but this increase was prevented when FOXL2 was silenced (Fig 1E). In contrast, SF-1 depletion did not significantly affect the capacity of FOXL2 to induce *AMH* transcriptional activation (Fig 1F).

### FOXL2 is required for the binding of SF-1 to the *AMH* promoter

**Fig 2:**
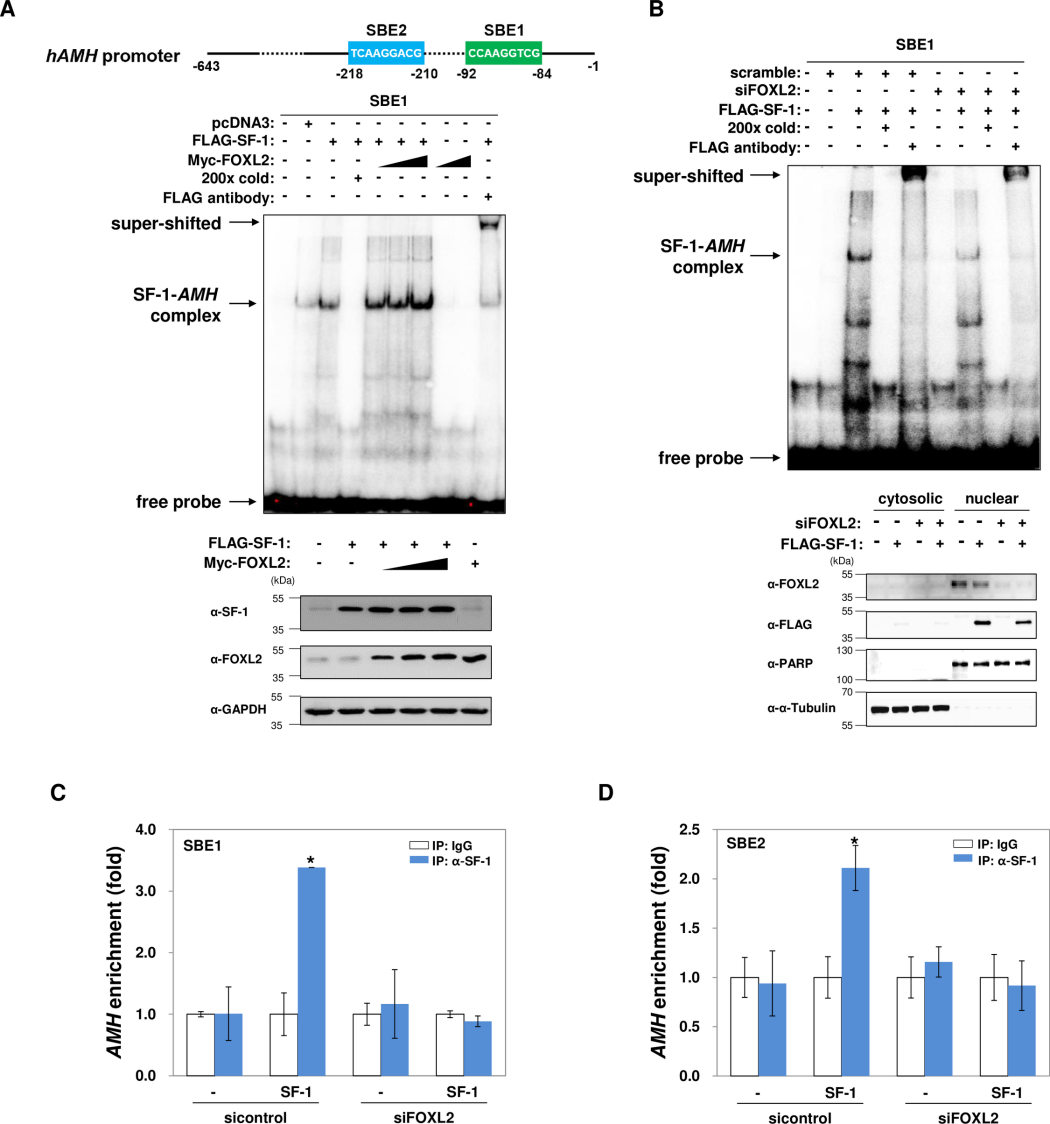
FOXL2 is essential for SF-1 association with *AMH*. (A) Two putative SF-1-binding elements (SBEs), SBE1 (-92/-84) and SBE2 (-218/-210), in the human *AMH* promoter are shown. (A–B) EMSA was performed using nuclear extracts (5 μg) from KGN cells transfected with the indicated plasmids or siRNAs. For the cold probe, a 200-fold excess of unlabeled oligonucleotides was used. The supershift of the band was determined by incubation of the nuclear extract with an anti-FLAG antibody. Radiolabeled-probes corresponding to the SBE1 sequences in the *AMH* promoter were used. Expression of SF-1 and FOXL2 proteins were determined by immunoblotting using the indicated antibodies (A; bottom panel). Efficient FOXL2 silencing and nuclear subcellular fractionation are shown in (B) (bottom panel). (C–D) KGN cells were transfected with the indicated plasmids and specific siRNAs for 24 h. Quantitative ChIP assays were performed using SF-1-specific primers that target SBE1 (C) or SBE2 (D), and quantitative real-time PCR results are shown as fold enrichment. As a negative control, control IgG was used for immunoprecipitation. Asterisks indicate significant values compared to those of controls. The results are from three independent experiments performed in duplicate (*p* <0.05).

To investigate how FOXL2 regulates SF-1-mediated *AMH* transcription, we determined if FOXL2 affected the binding of SF-1 to the *AMH* promoter. The human *AMH* promoter contains two SBEs at -92 to -84 (SBE1) and -218 to -210 (SBE2) (Fig 2A) [21]. Oligonucleotide probes specific for SBE1 were generated, and EMSA experiments on nuclear extracts from KGN cells overexpressing SF-1 and/or FOXL2 were performed. The results indicated the formation of a specific complex between SF-1 and the probe corresponding to SBE1, which was abolished or supershifted by the addition of the cold probe or antibody, respectively (Fig 2A). The formation of this complex, between SF-1 and *AMH*, was increased by FOXL2 overexpression (Fig 2A). In addition, the SF-1–*AMH* complex was greatly reduced in FOXL2-knockdown cells (Fig 2B). To further confirm the EMSA results, ChIP-qPCR experiments were performed. The promoter regions corresponding to SBE1 (Fig 2C) and SBE2 (Fig 2D) of endogenous *AMH* were significantly enriched in anti-SF-1 immunoprecipitates (Fig 2C and 2D). However, these enrichments were abolished in FOXL2-depleted KGN cells (Fig 2C and 2D), indicating an essential role for FOXL2 in the association between SF-1 and the *AMH* promoter. Similar results were observed in COV434 cells (S1A Fig).

**Fig 3:**
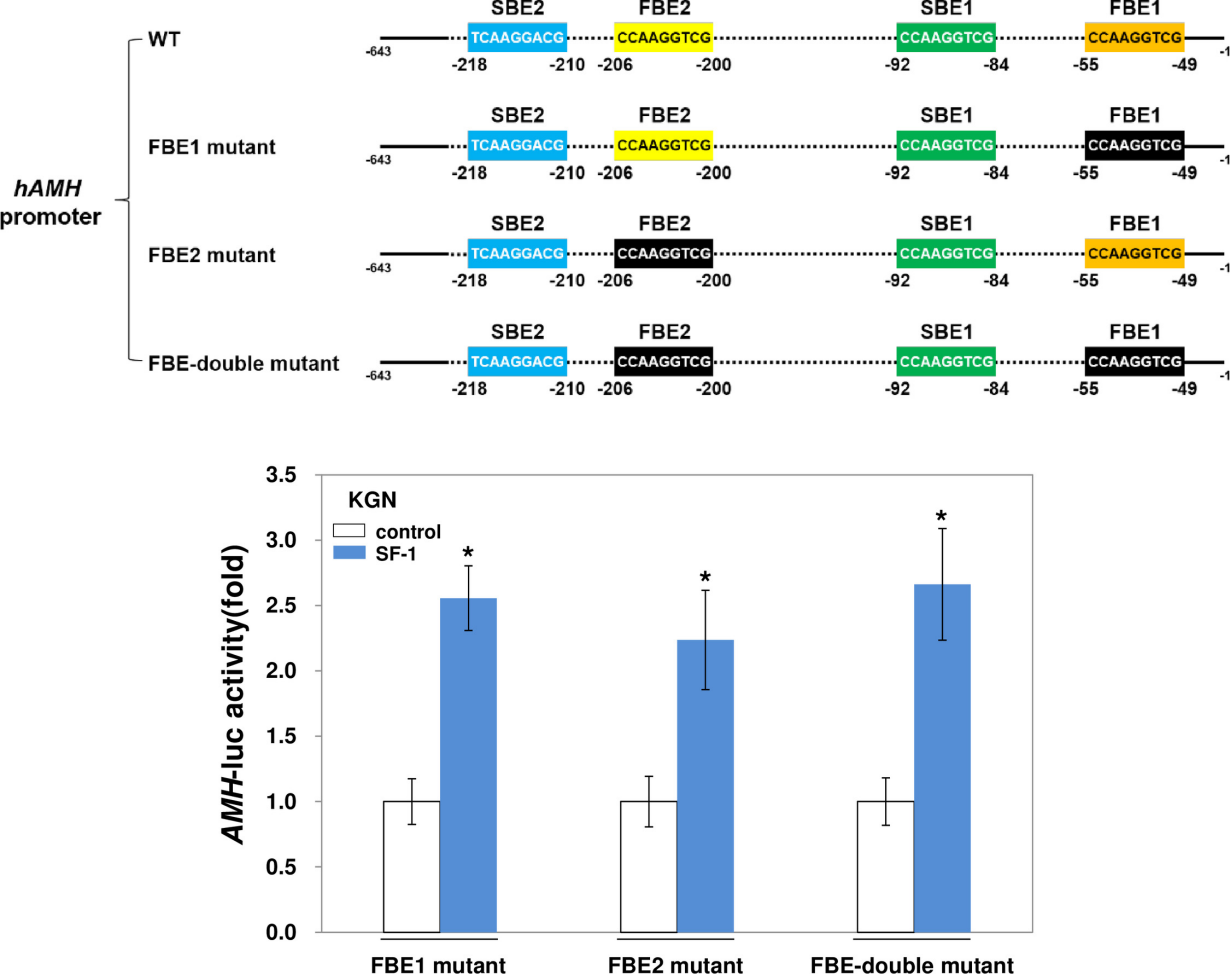
Transactivation of *AMH* by SF-1 does not require FOXL2-binding to the *AMH* promoter. *AMH* promoters with mutated FOXL2-binding elements (FBE1 and FBE2) are shown. Activation of the *AMH* promoter containing mutated FBEs (FBE1 mutant, FBE2 mutant, and FBE double mutant) by SF-1 was determined in KGN cells by luciferase assays. The luciferase activity was analyzed 24 h after transfection. Asterisks indicate significant values compared to control values. The results are from three independent experiments performed in duplicate (*p* < 0.05).

Because endogenous or overexpressed FOXL2 can also bind to the *AMH* promoter, at two forkhead-binding elements (FBEs) (S1B Fig) [29] located in the proximal vicinity of the SBEs (Fig 3), we determined whether FOXL2-association to *AMH* is prerequisite for SF-1-binding to *AMH*. Using *AMH* promoters with mutated FBEs to which FOXL2 is unable to bind [29], SF-1-induced reporter activities were measured. As shown in Fig 3, SF-1 was still able to activate these FBE mutated *AMH* promoters. Thus, this result indicates that the association of FOXL2 with *AMH* promoter is not necessary for SF-1-binding to *AMH*.

### The 290–291delCA-FOXL2 mutant fails to regulate SF-1-*AMH* binding due to defects in intracellular localization and interaction with SF-1

**Fig 4:**
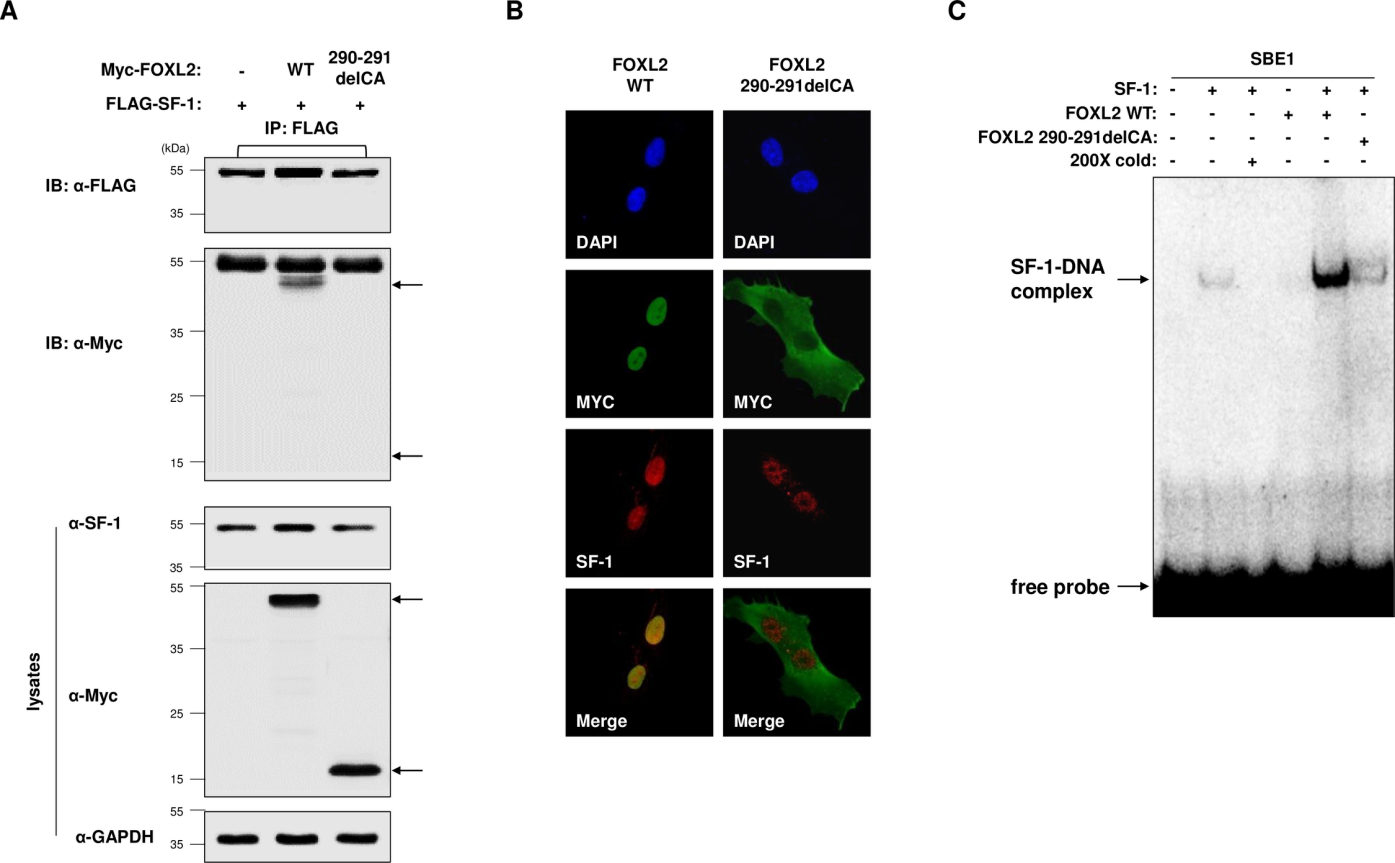
FOXL2–SF-1 binding is necessary for the association between SF-1 and the *AMH* promoter. (A) KGN cells were co-transfected with FLAG-SF-1 and Myc-tagged wild type (WT) FOXL2 or mutant FOXL2 (290–291delCA). After 24 h of incubation, cell lysates were prepared and immunoprecipitated with an anti-FLAG antibody. Immunoblot analyses were performed using the indicated antibodies. Arrows indicate the expected position of the proteins. (B) Myc-tagged WT and mutated FOXL2 were overexpressed in KGN cells; fluorescence confocal microscopy images are shown. (C) Purified recombinant SF-1 protein (0.2 μg) was incubated with WT or mutant FOXL2 proteins (0.2 μg) with radiolabeled, double-stranded oligonucleotides corresponding to the SBE1 of the *AMH* promoter.

We further investigated the possible mechanism for the defective transcriptional regulatory activity of mutant (290–291delCA) FOXL2 (observed in Fig 1B). Immunoprecipitation experiments to analyze protein–protein interactions revealed that the mutant could not bind to SF-1, whereas WT FOXL2 interacted with SF-1 (Fig 4A). In addition, mutant FOXL2 showed abnormal intracellular localization, as it was widely detected in the cytoplasm rather than in the nucleus; WT FOXL2 was mainly localized in the nucleus of KGN cells (Fig 4B). Moreover, EMSA using purified, recombinant proteins of WT FOXL2, mutant FOXL2, and SF-1 (S2 Fig), revealed that mutant FOXL2 failed to enhance SF-1 binding to the *AMH* promoter sequences (Fig 4C).

## Discussion

The development and growth of ovarian follicles, to generate competent oocytes, are complex processes that require highly ordered crosstalk with critical molecules and remains largely unspecified. Accumulating evidence implies that FOXL2, SF-1, and AMH could be important factors governing the ovarian follicle development. Herein, we provide, for the first time, experimental evidence that supports the presence of a coordinated regulatory network of FOXL2 and SF-1 on AMH production, which could be potentially important for controlling the ovarian folliculogenesis.

The ovarian reserve is comprised of a pool of resting primordial follicles and a pool of activated growing follicles. Approximately 680,000 non-growing follicles are endowed at birth, and follicles decline to 460,000 around puberty [32–35]. Monthly, cyclic recruitment of follicles continuously depletes the ovarian reserve until menopause [32, 35, 36]. Recently, serum AMH levels have gained attention as the most promising biomarker to accurately predict the ovarian reserve in clinical settings [37]. In addition, AMH measurement is accepted as a good method to predict the response to ovarian stimulation in assisted reproductive technology [38–40]. Although a decade has passed since the discovery of the inhibitory function of AMH on initial and cyclic ovarian follicle recruitments [2, 41, 42], the underlying regulatory mechanism of ovarian AMH production is still unclear.

Nuclear receptors are transcription factors that mostly function as homodimers or heterodimers [43]. SF-1 is a nuclear receptor acting as a monomer, and its function is regulated by binding to its interacting proteins [44]. Regarding *AMH* gene regulation, WT-1, DAX-1, SOX8, SOX9, GATA4, and NF-κB interact with SF-1 and positively or negatively regulate SF-1-induced *AMH* transcription during male sexual differentiation [17, 18]. Here, we first demonstrated that SF-1 is a transcriptional activator of ovarian *AMH* and further identified FOXL2 as an indispensable factor for SF-1-induced *AMH* regulation, based on the observation that SF-1 was unable to bind and transactivate the *AMH* promoter in the absence of a functional FOXL2 protein (Figs 1 and 2 and S1A Fig). The mechanism underlying this regulation by FOXL2 is likely to be through its interaction with SF-1 that subsequently allows association between SF-1 and SBEs in the human *AMH* promoter. Since the protein–protein interaction of SF-1 and FOXL2 involves their DNA-binding domains [30], it is plausible that the FOXL2 interaction at the DNA-binding domain of SF-1 may induce a conformational change in SF-1, particularly in the DNA-binding region, allowing SF-1 to bind to the *AMH* promoter. Meanwhile, as FOXL2 itself can bind to the *AMH* promoter, at two FBEs [29] located in the proximal vicinity of the SBEs (Fig 3), it is also possible that FOXL2 binding to *AMH* may alter the chromatin architecture, facilitating SF-1 access to SBEs on *AMH*. However, this is unlikely because SF-1 could still activate FBE-mutated *AMH* promoters (Fig 3), to which FOXL2 is unable to bind [29]. Therefore, the regulatory role of FOXL2 identified in this study is unique and critical because FOXL2 is an essential factor required for SF-1-induced *AMH* transcription in contrast to other SF-1-interacting proteins, such as WT-1, DAX-1, SOX8, SOX9, GATA4, and NF-κB, that merely function as modulators of SF-1-mediated transcriptional activation of *AMH*.

POI occurs by premature exhaustion of the ovarian reserve, leading to infertility, but its etiology is not well-defined because of the complex and heterogeneous nature of this condition [45, 46]. However, elucidation of ovarian AMH regulation at the molecular level can aid in understanding POI and normal ovarian folliculogenesis, based on the facts of the inhibitory role of AMH being demonstrated in follicle growth in mice [2, 41, 47] and extremely low or undetectable AMH levels in patients with POI [37]. A familial deletion mutant of FOXL2 (290–291delCA) that results in the production of a truncated FOXL2 protein lacking the forkhead DNA-binding domain was previously identified [31]. This mutant was employed in this study and can serve as a good model system, allowing us to gain molecular insights into POI, as the FOXL2 mutant was unable to interact with SF-1, failing to mediate the association between SF-1 and the *AMH* promoter. The inhibitory function of AMH on follicle recruitment [2, 41, 42] led us to speculate that the aberrant regulation of AMH results from loss-of-function mutations in FOXL2. This would result in insufficient production of AMH that would in turn lead to accelerated follicle growth, followed by premature exhaustion of the ovarian reserve. In addition to the activating role of FOXL2 in SF-1-induced *AMH* transcription identified in this study, FOXL2 itself transactivates *AMH* and the released AMH is further positively upregulated by FOXL2 [29]. Coinciding spatiotemporal expression of AMH, FOXL2, and SF-1 in granulosa cells of small follicles, and similar spectrums of ovarian phenotypes in mice with knockouts of these genes [27, 47, 48], also support the presence of this physiologically relevant regulatory network. Although more studies are needed for a complete understanding of AMH regulation and its molecular function in the ovary, this study demonstrates the presence of a critical regulatory network that could control ovarian folliculogenesis.

## Supporting Information

S1 Fig Transcriptional activation of *AMH* by SF-1 and FOXL2. Quantitative ChIP assays in COV434 (A) and KGN (B) cells were performed using SF-1-specific primers that target SBE1 or the FOXL2-specific primers for FOXL2 binding elements (FBEs) in the *AMH* promoter. (A) COV434 cells were transfected with control or SF-1 plasmids together with control siRNA or specific FOXL2 siRNA. (B) Enrichment of endogenous *AMH* and FOXL2 was determined by ChIP assay without transfection of plasmid in KGN cells. *AMH* luciferase activity was analyzed 24 h after transfection. Quantitative real-time PCR results are shown as fold enrichment. Control IgG was used for immunoprecipitation as a negative control. Asterisks indicate significant values compared with control values. The results are from three independent experiments performed in duplicate (*p* < 0.05).
(TIF)

S2 Fig Production and purification of recombinant proteins. The purity of (A) recombinant FLAG-tagged SF-1, (B) Myc-tagged WT FOXL2, and (C) Myc-tagged mutant FOXL2 (290–291delCA) proteins are demonstrated by coomassie blue staining. Arrows indicate the expected position of the proteins.
(TIF)
