# Supplementary figures and images for: FOXL2 Is an Essential Activator of SF-1-Induced Transcriptional Regulation of Anti-Müllerian Hormone in Human Granulosa Cells
Source: PLoS One. 2016 Jul 14;11(7):e0159112. doi: 10.1371/journal.pone.0159112 (PMC4944948; doi:10.1371/journal.pone.0159112)

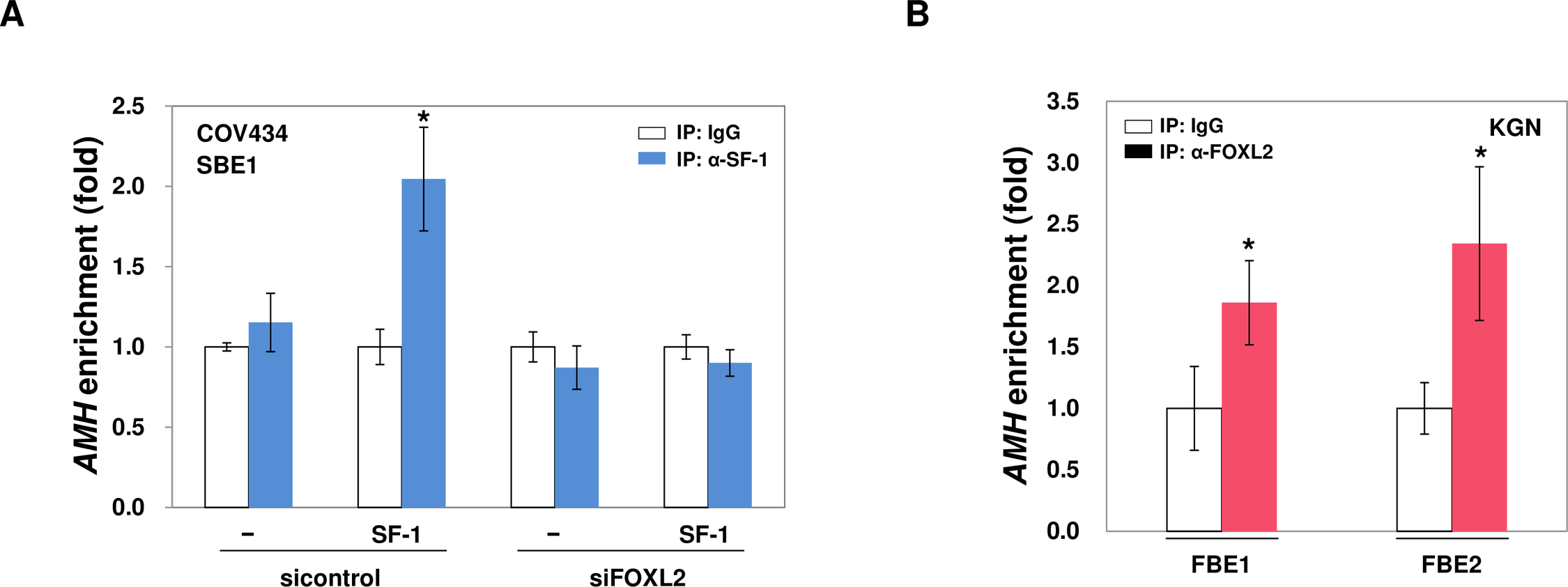

Supplement: S1 Fig — Quantitative ChIP assays in COV434 (A) and KGN (B) cells were performed using SF-1-specific primers that target SBE1 or the FOXL2-specific primers for FOXL2 binding elements (FBEs) in the AMH promoter. (A) COV434 cells were transfected with control or SF-1 plasmids together with control siRNA or specific FOXL2 siRNA. (B) Enrichment of endogenous AMH and FOXL2 was determined by ChIP assay without transfection of plasmid in KGN cells. AMH luciferase activity was analyzed 24 h after transfection. Quantitative real-time PCR results are shown as fold enrichment. Control IgG was used for immunoprecipitation as a negative control. Asterisks indicate significant values compared with control values. The results are from three independent experiments performed in duplicate (p < 0.05). (TIF) [file pone.0159112.s001.tif]

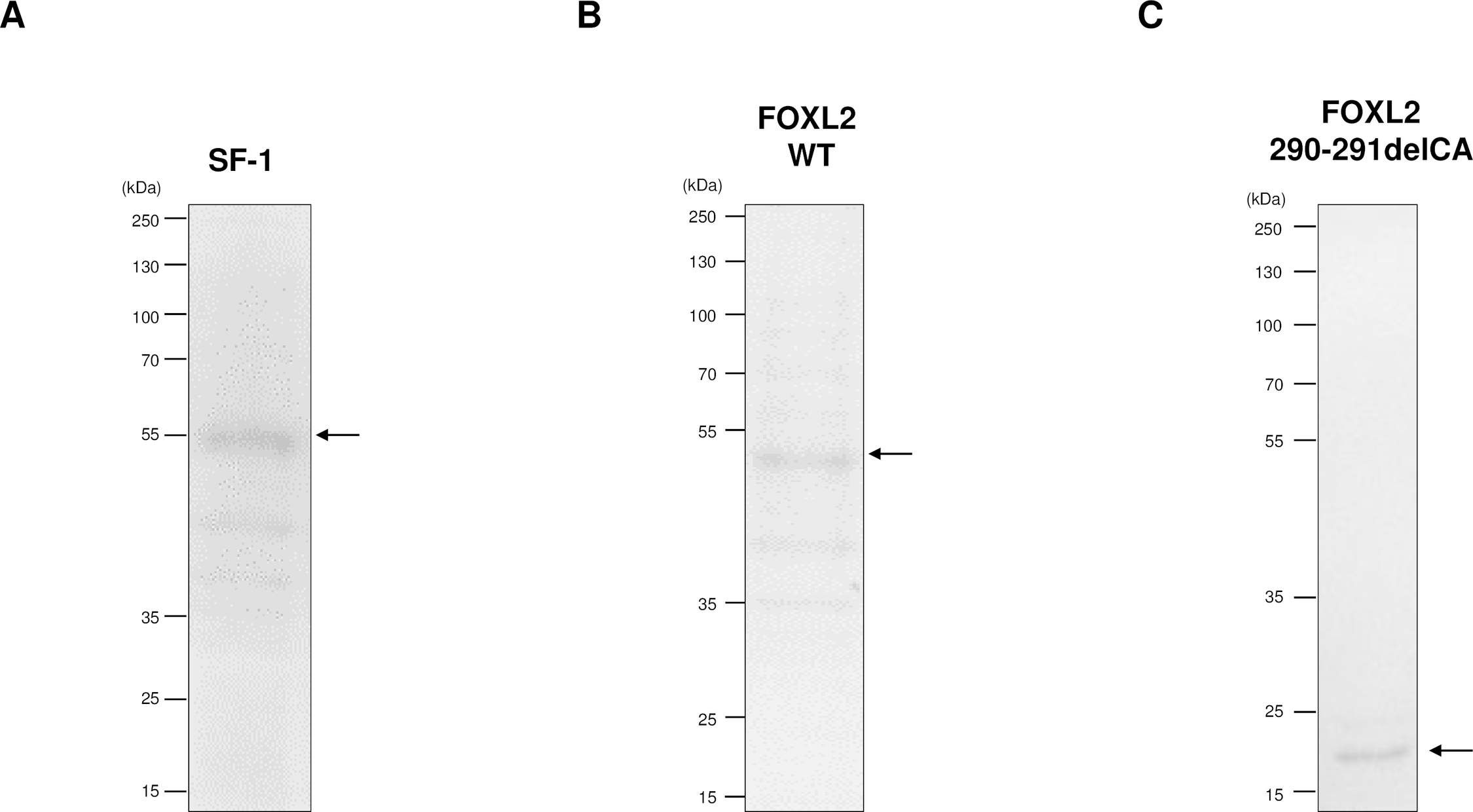

Supplement: S2 Fig — The purity of (A) recombinant FLAG-tagged SF-1, (B) Myc-tagged WT FOXL2, and (C) Myc-tagged mutant FOXL2 (290–291delCA) proteins are demonstrated by coomassie blue staining. Arrows indicate the expected position of the proteins. (TIF) [file pone.0159112.s002.tif]
